# A multi-omics approach identifies *bHLH71-like* as a positive regulator of yellowing leaf pepper mutants exposed to high-intensity light

**DOI:** 10.1093/hr/uhad098

**Published:** 2023-05-12

**Authors:** Zhoubin Liu, Lianzhen Mao, Bozhi Yang, Qingzhi Cui, Yunhua Dai, Xueqiao Li, Yisong Chen, Xiongze Dai, Xuexiao Zou, Lijun Ou, Sha Yang

**Affiliations:** Engineering Research Center of Education, Ministry for Germplasm Innovation and Breeding New Varieties of Horticultural Crops, Key Laboratory for Vegetable Biology of Hunan Province, College of Horticulture, Hunan Agricultural University, Changsha 410125, China; Engineering Research Center of Education, Ministry for Germplasm Innovation and Breeding New Varieties of Horticultural Crops, Key Laboratory for Vegetable Biology of Hunan Province, College of Horticulture, Hunan Agricultural University, Changsha 410125, China; Engineering Research Center of Education, Ministry for Germplasm Innovation and Breeding New Varieties of Horticultural Crops, Key Laboratory for Vegetable Biology of Hunan Province, College of Horticulture, Hunan Agricultural University, Changsha 410125, China; Engineering Research Center of Education, Ministry for Germplasm Innovation and Breeding New Varieties of Horticultural Crops, Key Laboratory for Vegetable Biology of Hunan Province, College of Horticulture, Hunan Agricultural University, Changsha 410125, China; Engineering Research Center of Education, Ministry for Germplasm Innovation and Breeding New Varieties of Horticultural Crops, Key Laboratory for Vegetable Biology of Hunan Province, College of Horticulture, Hunan Agricultural University, Changsha 410125, China; Institute of Vegetables, Hainan Academy of Agricultural Sciences, Haikou 570100, China; Institute of Vegetables, Hainan Academy of Agricultural Sciences, Haikou 570100, China; Engineering Research Center of Education, Ministry for Germplasm Innovation and Breeding New Varieties of Horticultural Crops, Key Laboratory for Vegetable Biology of Hunan Province, College of Horticulture, Hunan Agricultural University, Changsha 410125, China; Engineering Research Center of Education, Ministry for Germplasm Innovation and Breeding New Varieties of Horticultural Crops, Key Laboratory for Vegetable Biology of Hunan Province, College of Horticulture, Hunan Agricultural University, Changsha 410125, China; Engineering Research Center of Education, Ministry for Germplasm Innovation and Breeding New Varieties of Horticultural Crops, Key Laboratory for Vegetable Biology of Hunan Province, College of Horticulture, Hunan Agricultural University, Changsha 410125, China; Engineering Research Center of Education, Ministry for Germplasm Innovation and Breeding New Varieties of Horticultural Crops, Key Laboratory for Vegetable Biology of Hunan Province, College of Horticulture, Hunan Agricultural University, Changsha 410125, China

## Abstract

Light quality and intensity can have a significant impact on plant health and crop productivity. Chlorophylls and carotenoids are classes of plant pigments that are responsible for harvesting light energy and protecting plants from the damaging effects of intense light. Our understanding of the role played by plant pigments in light sensitivity has been aided by light-sensitive mutants that change colors upon exposure to light of variable intensity. In this study, we conducted transcriptomic, metabolomic, and hormone analyses on a novel yellowing mutant of pepper (*yl1*) to shed light on the molecular mechanism that regulates the transition from green to yellow leaves in this mutant upon exposure to high-intensity light. Our results revealed greater accumulation of the carotenoid precursor phytoene and the carotenoids phytofluene, antheraxanthin, and zeaxanthin in *yl1* compared with wild-type plants under high light intensity. A transcriptomic analysis confirmed that enzymes involved in zeaxanthin and antheraxanthin biosynthesis were upregulated in *yl1* upon exposure to high-intensity light. We also identified a single basic helix–loop–helix (bHLH) transcription factor, *bHLH71-like*, that was differentially expressed and positively correlated with light intensity in *yl1*. Silencing of *bHLH71-like* in pepper plants suppressed the yellowing phenotype and led to reduced accumulation of zeaxanthin and antheraxanthin. We propose that the yellow phenotype of *yl1* induced by high light intensity could be caused by an increase in yellow carotenoid pigments, concurrent with a decrease in chlorophyll accumulation. Our results also suggest that *bHLH71-like* functions as a positive regulator of carotenoid biosynthesis in pepper.

## Introduction

Plants use light receptors to perceive the intensity, direction, and photoperiod of light in order to properly regulate the timing of photomorphogenesis, flowering induction, circadian rhythm, and metabolism [1, 2]. In *Arabidopsis thaliana* (arabidopsis), light is thought to regulate expression of transporter genes by activating light-sensitive transcription factors that control the expression of genes involved in absorption and utilization of mineral elements [3]. The interacting proteins VASCULAR PLANT ONE-ZINC FINGER 1 (VOZ1) and VOZ2 promote flowering by inhibiting the expression of *FLC* (*FLOWERING LOCUS C*) [4]. Blue light promotes an association between the transcription factors CIB1 and CRY2 to form the Cry2–CIB1 complex, which binds the promoter of *FLOWERING LOCUS T* (*FT*) and drives *FT* expression to regulate flowering time [5].

Plant carotenoid metabolism is regulated by light [6]. Studies on yellowing maize seedlings have demonstrated that these plants respond rapidly to changes in light intensity by producing more carotenoid pigments through increased isopentenyl pyrophosphate isomerase activity in plastids [7]. The expression of phytoene synthase (*PSY*), which encodes an enzyme involved in carotenoid pigment production, is greater in white mustard seedlings exposed to light [8]. Carrots grown in light accumulate leaf-like carotenoids, whereas those grown in the dark accumulate mostly β-carotene [9]. Carotenoid accumulation in the chloroplasts of arabidopsis seedlings is caused by the light-induced expression of almost all genes in the mevalonic acid (MEP) metabolic pathway [10].

Plant color is primarily determined by the type, abundance, and ratio of pigments present in the tissue [11, 12], and photosynthetic pigments fall into one of two categories: chlorophyll or carotenoids [13]. Chlorophyll is the most important pigment in photosynthesis, as it is responsible for capturing light energy and transferring it to the photosystem reaction centers. Carotenoid is an umbrella term for carotene and xanthophylls, which are essential for photosynthesis and aid chlorophyll in capturing light and converting it into usable energy [14]. Studies have shown that leaf yellowing is primarily due to an inhibition of chlorophyll and carotenoid biosynthesis, which leads to reduced chlorophyll and carotenoid contents in yellowing varieties of plants [15]. Our understanding of light perception and responses in plants has been greatly aided by the availability of leaf color mutants in multiple plant species. These mutants have been used to better understand the photosynthetic system, chlorophyll metabolism, chloroplast development, and hormonal and metabolic responses to light [16, 17]. Their causal genes also serve as valuable markers used in genetic and breeding research for early screening of other important characteristics that are difficult to phenotype [18].

Research on leaf color mutants has revealed that chloroplast development and chlorophyll metabolism play an important role in color change. Several genes involved in chloroplast development and biosynthesis have also been characterized: *VIRESCENT-ALBINO LEAF 1* [19], *YLC2* [20], *WSL5* [21], and *CHLD* [22]. Although previous studies have shed light on the role of chlorophyll in leaf yellowing, less is known about the role played by carotenoids. This is despite the fact that the leaves, flowers, fruits, and roots of higher plants are rich in carotenoids that give them yellow, orange-red, or red colors [23, 24]. Carotenoid biosynthesis is affected by plant hormones, transcription factors, and plant developmental stage [25, 26]. Some transcription factors control carotenoid accumulation by regulating the expression of carotenoid-related genes. For example, silencing of *SlMBP8* significantly increased the expression of *PDS*, *PSY1*, and *ZDS* in tomato fruit, thereby increasing total carotenoid content [27]. Citrus *MYB68* can negatively regulate the expression of *BCH2* and *NCED5* and directly regulate the transformation of carotenoids from the α and β clades [28]. Basic helix–loop–helix (bHLH) transcription factors also participate in the regulation of plant carotenoid biosynthesis. The bHLH transcription factor *PIF1* inhibits transcription of *PSY* by binding to the G-box promoter element, thereby reducing carotenoid accumulation [29]. Overexpression of *SlPRE2* downregulated the chlorophyll biosynthesis genes *SlPSY1*, *SlPDS*, and *SlZDS* in tomato [30]. *CpbHLH1* and *CpbHLH2* in papaya can bind to *CpCYCB* and *CpLCYB* promoters to regulate carotenoid biosynthesis [31]. The phytohormones abscisic acid (ABA), auxin (IAA), brassinosteroids (BRs), ethylene (ETH), gibberellic acid (GA), and jasmonic acid (JA) also affect carotenoid accumulation during tomato fruit ripening [26, 32].

These studies clearly demonstrate that carotenoid accumulation in plants responds to changes in light intensity and hormone production. However, studies on leaf color mutants have overwhelmingly identified components of chlorophyll biosynthesis and not carotenoid biosynthesis. Therefore, the mechanistic regulation of carotenoid changes in leaf color mutants requires further study. Our laboratory previously used cobalt-60 radiation to generate a mutant population of pepper plants and isolated light-sensitive yellowing leaf mutants. Here, we analyzed the phenotypes of the *yl1* yellow-leaf mutant through hormone profiling and metabolomic and transcriptomic analyses. Our results revealed changes in the expression of genes related to carotenoid biosynthesis and hormone accumulation when the mutant was exposed to light of varying intensity. This study provides key insights into the molecular mechanism by which light affects carotenoid biosynthesis and provides a reference for crop improvement and leaf color research.

## Results

### Effects of light intensity on the phenotype of the *yellow leaf 1* (*yl1*) mutant

**Figure 1:**
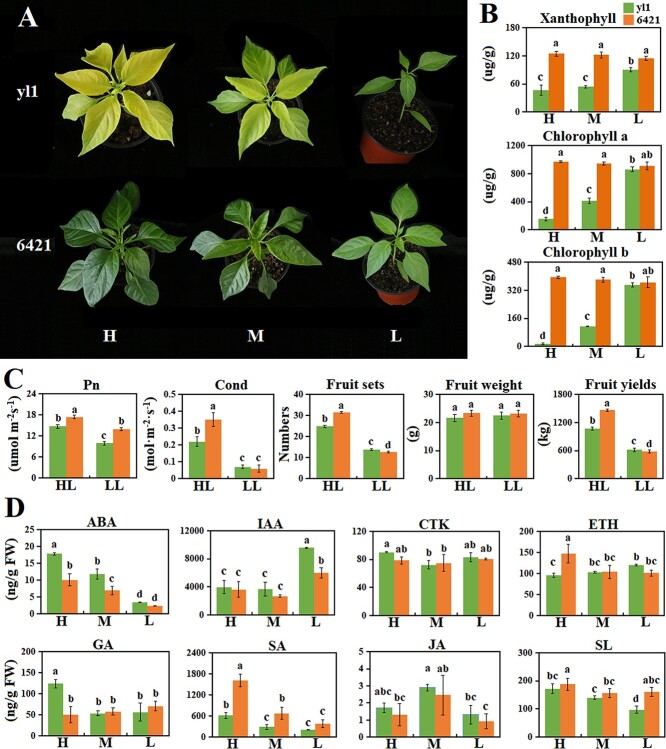
Phenotypes, agronomic traits, and hormone profiles of *yl1* and 6421 plants exposed to different light intensities. (**A**) Images of *yl1* and 6421 plants exposed to high (H), medium (M), or low (L) light conditions for 15 days. (**B**) Xanthophyll and chlorophyll contents in *yl1* and 6421 plants exposed to L, M, and H light for 15 days. (**C**) Net photosynthetic rate (Pn), stomatal conductance (Cond), fruit set per plant, average fruit weight, and total fruit yield of *yl1* and 6421 plants grown under field conditions with unfiltered light (HL) or 70% shade (LL). (**D**) Hormone concentrations of *yl1* and 6421 plants exposed to L, M, and H light for 15 days. Bars with different letters are significantly different (Duncan’s test; *P* < .05).

*Yellow leaf 1* (*yl1*) was identified from a population of mutagenized *Capsicum annuum* L. cultivar 6421 seedlings because its leaves became yellow upon exposure to light. To test the effects of different light intensities on the transition from green to yellow, we exposed *yl1* plants to high (500 μmol/m^2^/s), medium (200 μmol/m^2^/s), and low (50 μmol/m^2^/s) light intensities for 15 days. The leaves of *yl1* gradually changed from green to yellow as the light intensity increased, whereas wild-type plants (6421) remained green at all tested light intensities (Fig. 1A). The xanthophyll and chlorophyll contents were higher in leaves of 6421 than in those of *yl1* at all light intensities (Fig. 1B). In addition, there was a significant negative correlation between light intensity and the concentrations of these pigments in *yl1* leaves but not in 6421 leaves (Fig. 1B). A spectrophotometric analysis confirmed that the lightness (ΔL) and yellowness (Δb) values of *yl1* leaves were significantly higher than those of 6421 leaves and were positively correlated with light intensity (Table 1). Redness (Δa) was similar between the two pepper lines and showed little change, except under high light, when Δa was significantly higher in *yl1* leaves (Table 1). As a result, the total brightness value of *yl1* was significantly higher than that of 6421, especially under high light intensity. In summary, light intensity was positively correlated with the yellowness of *yl1* leaves, and the ratio of xanthophyll to chlorophyll also increased as light intensity increased.

**Table 1:** Color parameters of *yl1* and 6421 exposed to high (H), medium (M), or low (L) light conditions for 15 days. ΔL, color lightness value; Δa, redness value; Δb, yellowness value; ΔE, total chromaticity value. Values are means ± standard deviation from three experiments. Within a column, values with different lowercase letters are significantly different (Duncan’s test; *P* < .05)

	**Light intensity**	**ΔL**	**Δa**	**Δb**	**ΔE**
	H	67.89 ± 1.29 a	**+**0.03 ± 0.70 a	60.93 ± 1.87 a	91.22 ± 2.12 a
*yl1*	M	57.05 ± 2.11 b	−12.77 ± 0.33 d	44.17 ± 2.89 b	73.28 ± 3.34 b
	L	55.36 ± 2.22 b	−14.57 ± 0.18 e	41.96 ± 2.18 b	70.98 ± 2.94 b
	H	42.04 ± 1.27 d	−10.63 ± 0.27 b	21.17 ± 1.58 c	48.26 ± 1.84 c
6421	M	42.64 ± 1.37 cd	−11.97 ± 0.24 c	24.32 ± 1.32 c	50.53 ± 1.65 c
	L	43.00 ± 2.02 c	−12.98 ± 0.48 d	26.60 ± 2.90 c	52.22 ± 3.26 c

We next quantified multiple agronomic traits of *yl1* and 6421 grown in the field with unfiltered light or 50% shade. The net photosynthetic rate (Pn), number of fruits per plant, and fruit yield were significantly lower in *yl1* than in 6421 under normal light conditions in the field (Fig. 1C). Interestingly, although the Pn of *yl1* grown in 70% shade was still significantly lower than that of 6421, the number of fruits set and the yield were higher in *yl1* than 6421 under these lower light conditions (Fig. 1C).

### Effects of light intensity on hormone production in *yl1*

Carotenoids are precursors for the biosynthesis of strigolactones (SLs) and ABA [33], and changes in carotenoid accumulation caused by changes in light intensity may alter the abundance of these plant hormones. The concentrations of 36 hormones from eight classes were quantified in the *yl1* and 6421 pepper lines upon exposure to high, medium, and low light for 15 days (Supplementary Data Fig. S1). When hormones were grouped by class, ABA and salicylic acid (SA) accumulation were positively correlated with light intensity in both plant lines, whereas IAA production was elevated under low light in both plant lines (Fig. 1D). In general, ABA concentration was significantly higher in *yl1* than 6421, whereas SA concentration was higher in 6421 (Fig. 1D). Light intensity had little effect on CK, ETH, and GA concentrations in either line (Fig. 1D). Gibberellic acid concentration was significantly higher in *yl1* only under high light, but there was a weak positive correlation between SL accumulation and light intensity in both lines (Fig. 1D).

### Effects of light intensity on carotenoid accumulation in *yl1*

Changes in lutein content in *yl1* plants during yellowing led us to speculate that other carotenoids may also differentially accumulate upon changes in light intensity. To better understand how light intensity affects the carotenoid metabolic pathway, we analyzed the carotenoid content of *yl1* and 6421 using LC–MS/MS. A total of 28 carotenoids were detected in both lines upon exposure to high, medium, and low light. Antheraxanthin, α-carotene, β-carotene, β-cryptoxanthin, lutein, neoxanthin, phytoene, violaxanthin, and zeaxanthin were abundant, whereas levels of the other 20 carotenoids were low (Supplementary Data Table S1). In 6421, no significant differences in accumulation of phytoene, phytofluene, lutein laurate, antheraxanthin, zeaxanthin, lutein, neoxanthin, and β-carotene were observed upon exposure to any of the light intensities (Supplementary Data Table S1). However, in *yl1*, light intensity was significantly and positively correlated with accumulation of phytoene, phytofluene, lutein laurate, antheraxanthin, and zeaxanthin, and accumulation of these carotenoids was significantly higher in *yl1* than in 6421 (Supplementary Data Table S1). Lutein, neoxanthin, and β-carotene accumulation were negatively correlated with light intensity in *yl1*, but no significant difference was observed under low light in 6421 (Supplementary Data Table S1).

### Differentially expressed genes in *yl1* and 6421 exposed to high, medium, and low light

**Figure 2:**
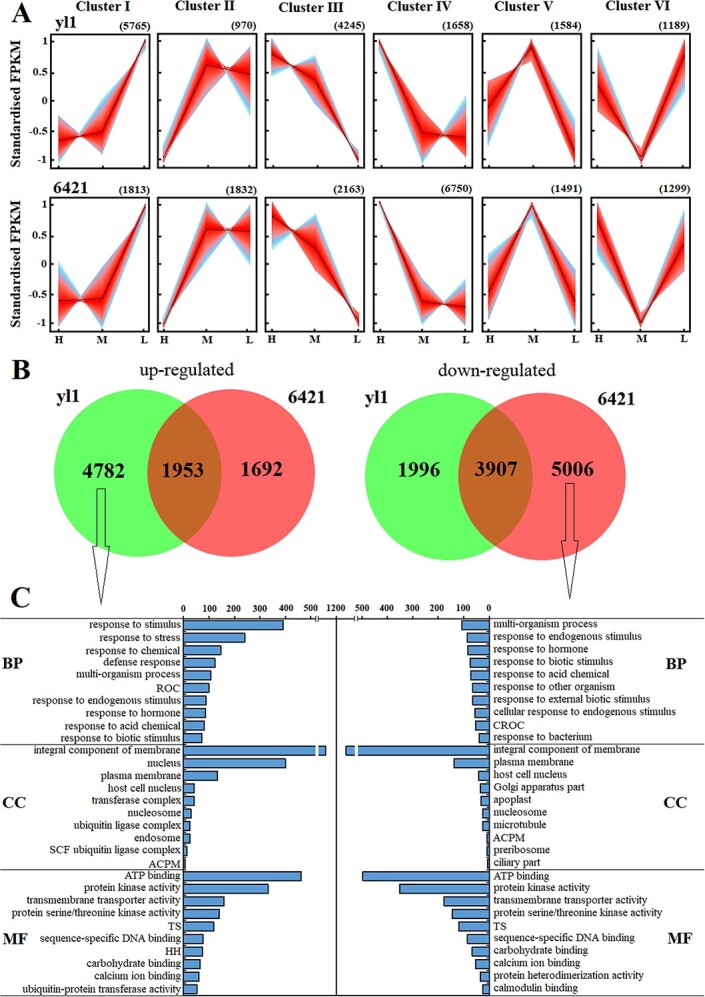
. DEGs in *yl1* and 6421 exposed to high, medium, or low light for 15 days. (**A**) Unsupervised fuzzy clustering of DEGs (numbers in parentheses are the total numbers of DEGs in each cluster). (**B**) Venn diagram of DEGs. (**C**) GO enrichment analysis of DEGs, showing biological process (BP), cellular component (CC), and molecular function (MF) terms. ROC, response to oxygen-containing compound; ACPM, anchored component of plasma membrane; HH, hydrolase activity, hydrolyzing *O*-glycosyl compounds; TS, transcription factor activity, sequence-specific DNA binding; CROC, cellular response to oxygen-containing compound.

We next performed RNA-seq to analyze the effect of light intensity on expression of carotenoid biosynthesis-related genes in *yl1* and 6421 plants exposed to high, medium, or low light for 15 days. Approximately 381 million clean, high-quality reads were obtained using the Illumina NovaSeq 6000 system. Reads were mapped to the *C. annuum* L*_*Zunla-1 reference genome, and 19 312 differentially expressed genes (DEGs) were identified out of 44 971 expressed genes (Supplementary Data Table S2). We performed unsupervised fuzzy clustering of all DEGs to obtain an unbiased assessment of gene expression dynamics under different light intensities. We identified six DEG clusters that could be further divided on the basis of three expression trends: a positive correlation between gene expression and light intensity (clusters III and IV), a negative correlation between gene expression and light intensity (clusters I and II), and similar expression in high and low light (clusters V and VI) (Fig. 2A).

To better understand the difference in light response between *yl1* and 6421, we performed a Gene Ontology (GO) term analysis on DEGs from clusters I, II, III, and IV because the DEGs in these clusters clearly responded to light intensity. We compared 4782 DEGs that were specifically upregulated in *yl1* in response to increasing light intensity with 5006 DEGs that were specifically downregulated in 6421 in response to increasing light intensity (Fig. 2B). Enriched GO terms shared between the upregulated genes in *yl1* and the downregulated genes in 6421 included ATP binding, integral component of membrane, plasma membrane, protein kinase activity, and transmembrane transporter activity (Fig. 2C). Genes related to these processes and cellular compartments may be involved in the leaf yellowing of *yl1* in response to light.

### Differential expression of genes in the carotenoid pathway

**Figure 3:**
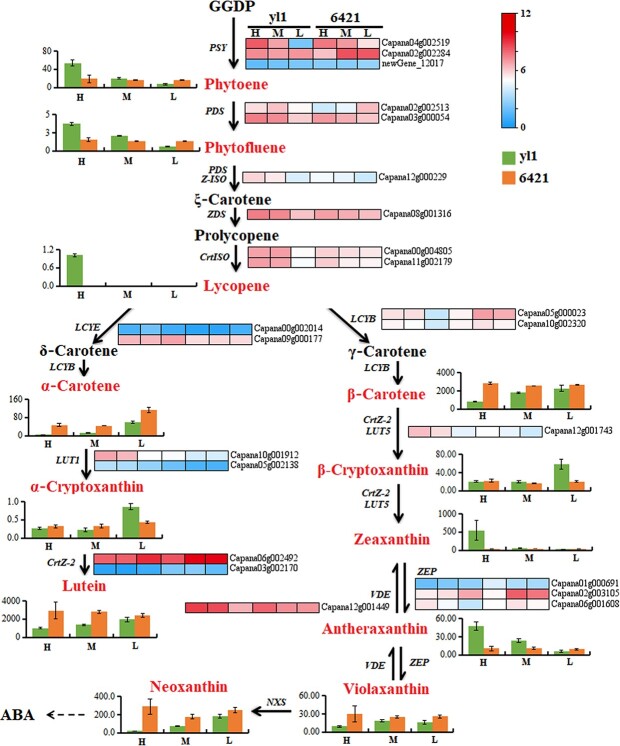
. Comparative analysis of genes and metabolites related to carotenoid biosynthesis in *yl1* and 6421 plants exposed to high (H), medium (M), or low (L) light intensity. CrtISO, carotene isomerase; CrtZ-2, β-carotene hydroxylase 2; GGDP, geranylgeranyl diphosphate; LCYB, lycopene β-cyclase; LCYE, lycopene ε-cyclase; LUT1, carotenoid epsilon hydroxylase; LUT5, β-ring hydroxylase; NXS, neoxanthin synthase; PDS, phytoene desaturase; PSY, phytoene synthase; VDE, violaxanthin de-epoxidase; ZDS, ζ-carotene desaturase; Z-ISO, ζ-carotene isomerase. Metabolites are in red text and were quantified using LC–MS/MS. Bar graphs show metabolite content (μg/g) and heat maps show gene expression data.

There were more DEGs in the carotenoid biosynthesis pathway in *yl1* plants than in 6421 plants, suggesting that carotenoid biosynthesis is more sensitive to changes in light intensity in *yl1* (Fig. 3). The expression levels of *PSY* (*Capana04g002519*) and *PDS* (*Capana03g000054*), two genes required for carotenoid biosynthesis, were significantly lower in *yl1* compared with 6421 under low-intensity light but not under high-intensity light (Fig. 3). This reduction in *PSY* and *PDS* expression in low-intensity light may explain the significantly lower phytoene and phytofluene accumulation in *yl1*. The expression levels of *carotene isomerase* (*CrtIS*, *Capana00g004805* and *Capana11g002179*) and *β-ring hydroxylase* (*LUT5*, *Capana12g001743*) in *yl1* were positively correlated with light intensity and coincided with a large increase in lycopene and zeaxanthin accumulation (Fig. 3). *β-Carotene hydroxylase 2* (*CrtZ-2*, *Capana06g002492*) and *Lycopene β-cyclase* (*LCYB*, *Capana05g000023*) expressions were negatively correlated with light intensity in *yl1* and 6421. Interestingly, *CrtZ-2* expression was not significantly different between *yl1* and 6421 under low light, and *LCYB* expression was not significantly different between *yl1* and 6421 under high light; however, *LCYB* expression did decrease 1.76-fold in *yl1* and increase 1.85-fold in 6421 under low light compared with high light (Fig. 3). We also noticed that *zeaxanthin de-epoxidase* (*ZEP*, *Capana02g003105*) and *violaxanthin de-epoxidase* (*VDE*, *Capana12g001449*) expression were inversely related in *yl1*: *ZEP* expression was negatively correlated with light intensity, whereas *VDE* expression was positively correlated with light intensity (Fig. 3). This expression pattern may promote the synthesis and accumulation of zeaxanthin and antheraxanthin in *yl1* under high light intensity and result in leaf yellowing.

### Expression of bHLH transcription factors

**Figure 4:**
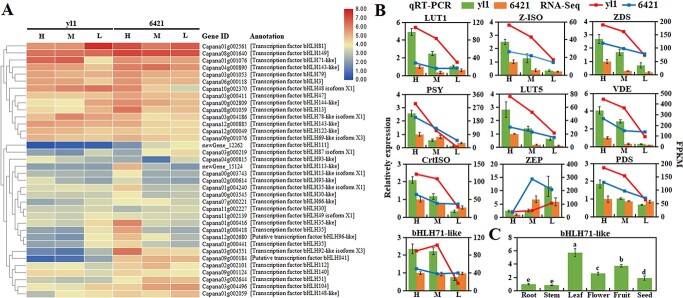
. Expression of bHLH transcription factor genes in *yl1* and 6421 exposed to high (H), medium (M), or low (L) light intensity. (**A**) Heat map of bHLH transcription factor expression. (**B**) qRT–PCR validation of carotenoid-related and *bHLH71-like* transcription factor genes. (**C**) Expression of *bHLH71-like* genes in different pepper tissues measured by qRT–PCR.

Carotenoid pathways are regulated in part by bHLH transcription factors in plants [29–31, 34], and many genes encoding bHLHs were identified in the transcriptomes of *yl1* and 6421 leaves (Supplementary Data Table S3). We identified 36 bHLH transcription factors with an FPKM (fragments per kilobase of transcript per million mapped reads) value >10 in at least one combination of genotype and light treatment (Fig. 4A). However, only *bHLH81* (*Capana01g002561*) and *bHLH71-like* (*Capana01g001076*) were differentially expressed between *yl1* and 6421. The expression level of *bHLH81* in *yl1* was negatively correlated with light intensity. However, the expression level of *bHLH71-like* in *yl1* was positively correlated with light intensity but remained stable and lower in 6421. The expression levels of select carotenoid biosynthesis-related genes and bHLH transcription factor genes were validated by qRT–PCR (Fig. 4B). An analysis of *bHLH71-like* expression in different pepper plant tissues revealed that expression was highest in leaf tissue followed by fruit and flower tissue. The lowest expression levels were found in roots and stems (Fig. 4C).

### Subcellular localization and gene interactions of *bHLH71-like* transcription factors

**Figure 5:**
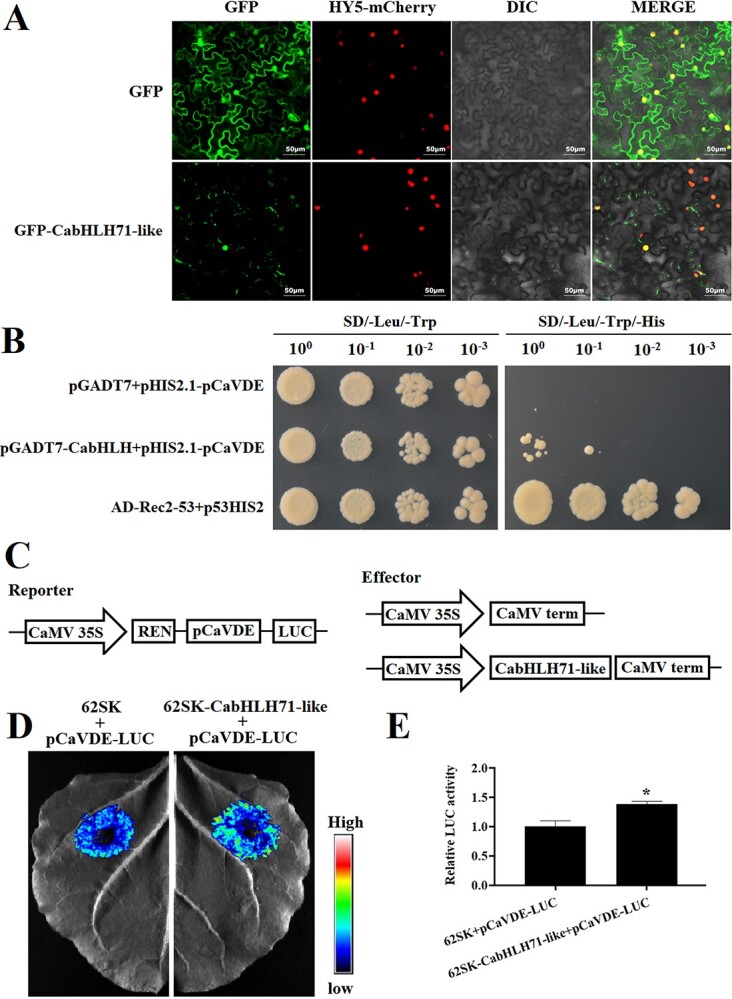
. Subcellular localization of *bHLH71-like* and promoter binding analysis. (**A**) The nuclear marker *HY5-mCherry* was co-expressed with *GFP-bHLH71-like* in *N. benthamiana* leaf epidermal cells, and fluorescence microscopy was performed to determine whether the mCherry and GFP signals colocalized. GFP fluorescence, mCherry fluorescence, differential interference contrast (DIC), and merged images are shown. Three independent experiments were performed with similar results. Bar, 50 μm. (**B**) Yeast one-hybrid assay showing that *bHLH71-like* binds to the *pCaVDE* promoter. Serially diluted cell suspensions (OD_600_ = 0.2, 0.02, 0.002, and 0.0002) were spotted onto selective (SD/−Leu/−Trp/−His) or non-selective (SD/−Leu/−Trp) medium. AD-REC2-P53 and p53HIS2 were used as a positive control and AD+pHIS2.1-p*CaVDE* as a negative control. (**C**) Reporter and effector vectors used in the dual-luciferase assay. (**D**) LUC images of tobacco leaves after transient infiltration. (**E**) Ratio of LUC to REN activity. Data are means ± standard deviation from at least three biological replicates. The asterisk indicates a significant difference relative to the vector control (Duncan’s test, ^*^P < .05).

The difference in *bHLH71-like* expression between *yl1* and 6421, along with the high expression of this gene in leaves, led us to hypothesize that it may play a role in regulating carotenoid metabolism in *yl1* leaves under conditions of low light intensity. As a putative transcription factor, we anticipated that *bHLH71-like* would localize to the nucleus. Upon co-expression of *GFP*-*bHLH71-like* and the nuclear marker *AtHY5-mCherry* in *Nicotiana benthamiana* leaves, we observed a clear overlap in GFP and mCherry fluorescence (Fig. 5A). Previous studies have demonstrated that bHLH transcription factors modulate the transcription of carotenoid biosynthesis genes by binding to G-box elements in their promoters [35]. Because the *CaVDE* promoter contains a G-box element, we performed a yeast one-hybrid assay to determine whether *bHLH71-like* could regulate *CaVDE* expression. The results showed that *bHLH71-like* could bind the promoter of *CaVDE* to drive expression of the reporter and support yeast growth on minimal medium (Fig. 5B). A dual luciferase assay confirmed the ability of bHLH71-like to bind the *CaVDE* promoter and drive *luciferase* expression (Fig. 5C–E).

### Phenotypic analysis and changes in gene expression in *CaVDE-* and *bHLH71-like*-silenced plants

**Figure 6:**
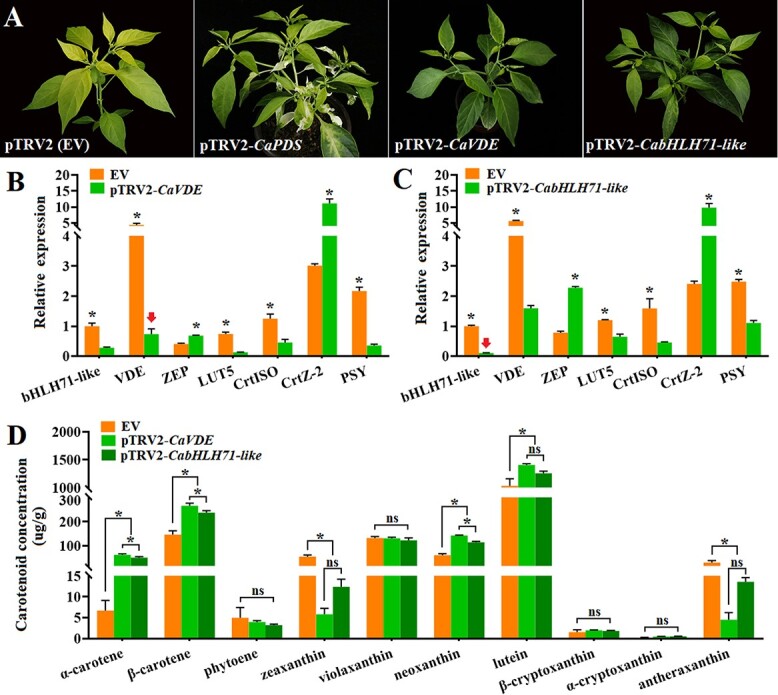
. Carotenoid-related gene expression and metabolite concentrations in *yl1* plants with reduced expression of *CaVDE* and *bHLH71-like*. (**A**) Phenotypes of *yl1* plants inoculated with an empty vector (EV) VIGS construct and constructs targeting *PDS*, *CaVDE*, and *bHLH71-like*. (**B**) Relative expression levels of carotenoid-related genes in *CaVDE*-silenced plants. (**C**) Relative expression levels of carotenoid-related genes in *bHLH71-like*-silenced plants. (**D**) Concentrations of carotenoid metabolites. Error bars represent standard deviations. Asterisks indicate significant differences (Duncan’s test, ^*^P < .05). ns, no significant difference.

To determine whether elevated levels of zeaxanthin and antheraxanthin in *yl1* exposed to high light intensity could cause the observed yellow phenotype, we silenced *CaVDE* and *bHLH71-like* in *yl1* using virus-induced gene silencing (VIGS). The results demonstrated that silencing of *CaVDE* or *bHLH71-like* suppressed the yellowing phenotype (Fig. 6A). The expression levels of *CaVDE* and *bHLH71-like* were significantly downregulated in the silenced lines, confirming that silencing of both genes using VIGS was successful (Fig. 6B and C). We also found that other genes involved in carotenoid metabolism showed the same expression trend in both *CaVDE-* and *bHLH71-like*-silenced lines. The expression levels of *CaZEP* and *CaCrtZ-2* were significantly increased, whereas *CaLUT5*, *CaCrtISO*, and *CaPSY* expression levels were significantly decreased.

### Changes in carotenoid accumulation in *CaVDE-* and *bHLH71-like*-silenced plants

Changes in the expression of carotenoid biosynthesis-related genes in the *CaVDE-* and *bHLH71-like-*silenced *yl1* plants suggested that carotenoid accumulation may be affected in these plants. We used HPLC–mass spectrometry to measure the concentrations of key metabolites in the carotenoid biosynthesis pathway in *CaVDE-* and *bHLH71-like-*silenced lines (Fig. 6D). Accumulation of α-carotene, β-carotene, neoxanthin, and lutein was significantly higher when *CaVDE* and *bHLH71-like* were silenced, whereas zeaxanthin and antheraxanthin accumulation was significantly lower (Fig. 6D). These results suggest that *CaVDE* positively regulates the yellowing response in *yl1* and that silencing of *CaVDE* in *yl1* maintains green leaves under high light intensity by reducing zeaxanthin and antheraxanthin accumulation. These results also confirmed that *bHLH71-like* can regulate the expression of carotenoid biosynthesis genes such as *CaVDE* in response to light intensity, thus regulating the metabolism of carotenoids in pepper leaves and controlling the leaf color of *yl1*.

## Discussion

Carotenoids are important light-harvesting pigments that can be divided into two groups: carotenes and xanthophylls. The xanthophyll cycle lies downstream of the carotenoid biosynthesis pathway and is believed to be the major pathway that protects plants against stress damage caused by high light intensity [36–38]. Violaxanthin de-epoxidase and ZEP are key enzymes that regulate the xanthophyll cycle [39] and catalyze the mutual transformations among antheraxanthin, violaxanthin, and zeaxanthin. In excessive light, VDE catalyzes violaxanthin de-epoxidation to form antheraxanthin. Subsequent de-epoxidation of antheraxanthin by VDE then forms zeaxanthin, which provides photoprotection by quenching excited chlorophyll molecules and changing the fluidity of thylakoid membranes to dissipate excess light energy [40]. By contrast, ZEP catalyzes the reverse reactions under low light intensity to regenerate violaxanthin from zeaxanthin and antheraxanthin [41]. We observed reduced expression of *ZEP* in *yl1* plants exposed to high light intensity, which corresponded with the increased accumulation of zeaxanthin (Fig. 3). Increased *ZEP* expression occurred under low light intensity, which corresponded to a decrease in zeaxanthin accumulation and an increase in violaxanthin accumulation (Fig. 3). As expected, the expression pattern of *VDE* was opposite to that of *ZEP* in *yl1*, which likely contributed to the balance between zeaxanthin and violaxanthin at high and low light intensities (Fig. 3). Silencing *VDE* in *yl1* plants rescued the yellowing phenotype under high light intensity and resulted in decreased accumulation of zeaxanthin and antheraxanthin (Fig. 6). These results suggest that yellowing in *yl1* is likely the result of increased accumulation of antheraxanthin and zeaxanthin.

As well as being produced by the xanthophyll cycle, zeaxanthin can also be produced from β-carotene under high light intensity in a reaction catalyzed by LUT5. Our results demonstrated that *LUT5* expression was significantly increased in *yl1* leaves under high versus low light intensity (Fig. 3). The expression patterns of *ZEP*, *VDE*, and *LUT5* in *yl1* under high light intensity, together with the observed increases in zeaxanthin and antheraxanthin concentrations, suggest that the yellowing phenotype of *yl1* is due to increased accumulation of zeaxanthin and antheraxanthin. This could serve as an adaptive response to maintain normal photosynthetic capacity and efficiency under high light intensity. The *yl1* mutant can better dissipate excessive heat in field conditions with high light intensity by reducing its net photosynthetic rate. However, the cost of this response is a lower yield than that of wild-type plants (Fig. 1C).

Light intensity is known to regulate the accumulation of important plant hormones, such as GA, ABA, IAA, and SA [42–47]. ABA and GA co-regulate stomatal opening and closing in response to light conditions [43]. SA has a protective effect on the photosystem II (PS-II) reaction center, which enables PS-II to maintain high optical activity, promotes the opening of stomata in leaves, and improves photosynthetic efficiency [46]. We observed increased accumulation of ABA, SA, and GA in *yl1* under high light intensity, whereas IAA accumulation was decreased (Fig. 1D). Changes in these hormone levels promoted more open stomata in the *yl1* mutant under high light intensity compared with low light intensity (Fig. 1C). This resulted in higher PS-II optical activity, which allowed *yl1* plants to maintain normal photosynthesis under high light intensity and avoid light-induced damage. In addition, IAA accumulation decreased significantly under high light intensity. This is consistent with previous studies on IAA accumulation in arabidopsis subjected to continuous light [45]. The reason for this decrease is that IAA is easily oxidized and degraded under high light [44].

Members of the bHLH transcription factor family have previously been suggested to regulate plant carotenoid biosynthesis. Genes known to be negatively regulated by bHLH transcription factors include *PSY*, *PDS*, and *ZDS* [29, 30, 34, 48]. Our transcriptomic comparison between *yl1* and 6421 plants revealed that a single bHLH transcription factor, *bHLH71-like*, was differentially expressed and positively correlated with light intensity in *yl1* (Fig. 4). Silencing *bHLH71-like* in the *yl1* background resulted in reduced expression of *PSY*, *VDE*, *CrtISO*, and *LUT5* under high light intensity (Fig. 4B). Downregulation of these genes also coincided with a significant reduction in zeaxanthin and antheraxanthin accumulation and leaves that remained green in the *yl1* line when exposed to high light intensity (Fig. 6A). These results contrast with previous studies demonstrating that carotenoid biosynthesis-related genes are typically negatively regulated by bHLH transcription factors [29, 30, 34, 48]. In addition to our findings, it has also been reported that *CpbHLH2* functions as an activator of *LCYB* expression in papaya [31]. Therefore, future work may uncover additional bHLH transcription factors that function as positive regulators of carotenoid biosynthesis. These insights could then be used to enhance light sensitivity/tolerance in pepper plants and other crop species through genetic modification or engineering of this large and highly conserved family of proteins.

### Conclusions

**Figure 7:**
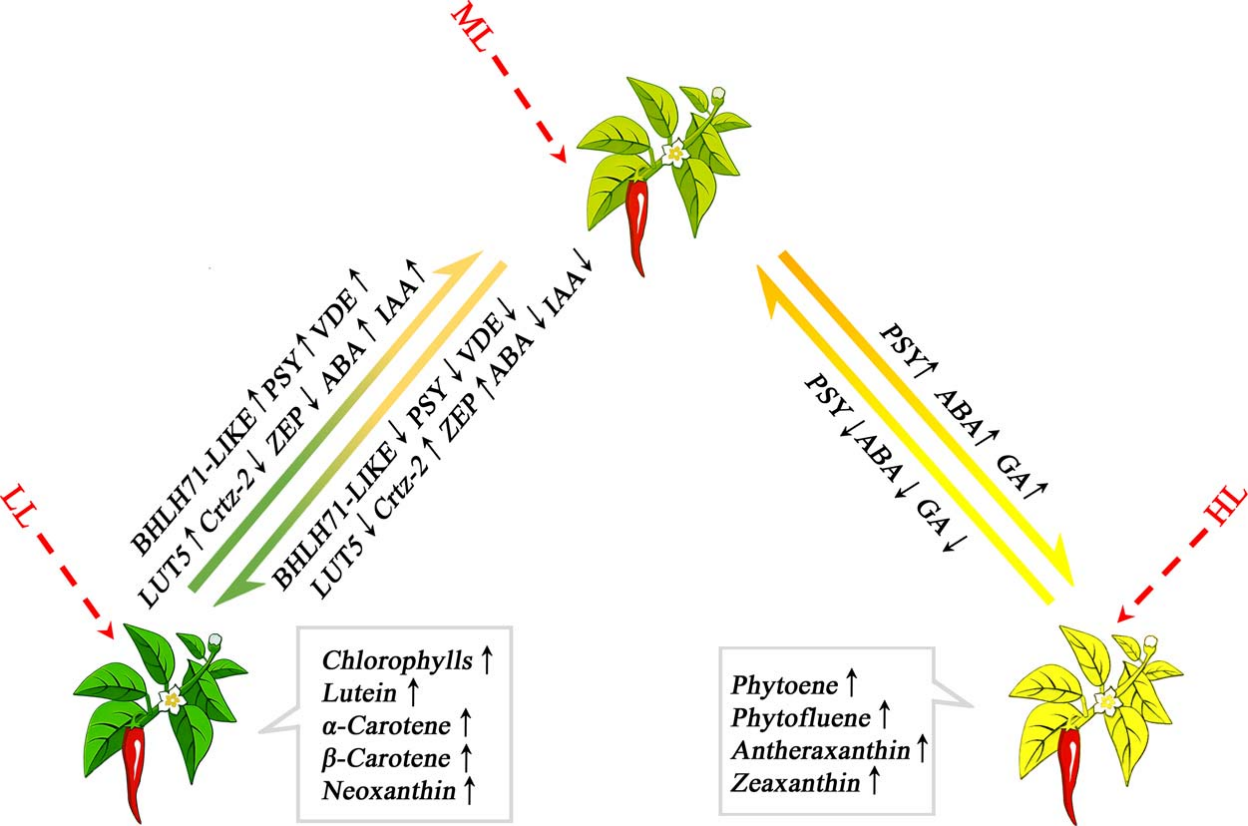
Schematic diagram of the regulatory network that controls the yellowing phenotype in *yl1* plants under high-intensity light. Arrows beside gene and metabolite names indicate increases (upward) or decreases (downward) in gene expression and metabolite content. The red dotted arrows represent light conditions. HL, high light. ML, medium light. LL, low light.

Transcriptomic, metabolomic, and hormonal analyses were used to investigate the molecular mechanism of yellowing in the *yl1* pepper mutant exposed to high-intensity light. Based on our results, we propose a model for light-induced yellowing in the *yl1* mutant (Fig. 7). In this model, the balance between chlorophyll and carotenoid accumulation changes at different light intensities. Chlorophyll content tends to decrease under high light intensity, whereas phytoene, phytofluene, and lycopene tend to accumulate. Increased expression of *bHLH71-like*, *CaVDE*, and *LUT5* and reduced expression of *ZEP* further promote the biosynthesis and accumulation of antheraxanthin and zeaxanthin. Taking these results together, these changes are responsible for yellowing in *yl1* plants exposed to high-intensity light. Under low light intensity, *CrtZ-2* and *ZEP* expression is high, whereas *bHLH71-like*, *CaVDE*, and *LUT5* expression is low. This leads to significantly reduced accumulation of antheraxanthin and zeaxanthin, whereas lutein accumulation increases. Finally, we found that the yellowing phenotype of *yl1* plants subjected to high light intensity could be suppressed through silencing of *bHLH71-like* and *CaVDE.*

## Materials and methods

### Planting and sampling

The *yl1* mutant and 6421 wild-type pepper lines were provided by the Pepper Research Group of Hunan Agricultural University. Pepper seeds were sown in 50-hole trays containing a nutrient substrate with an organic matter content of ≥20% and a pH of 5.5–7. Once seedlings had developed four leaves, they were transplanted into 6 × 10-cm pots and grown under high (500 μmol/m^2^/s), medium (200 μmol/m^2^/s), or low (50 μmol/m^2^/s) light intensity in a controlled illumination incubator (HP600GS-LED, Jingsheng Scientific Instrument, Shanghai). Plants were grown under a 16-hour light (28°C)/8-hour dark (20°C) photoperiod at a constant relative humidity of 65 ± 5%. There were three replicates of each light treatment, each with 15 seedlings per genotype. After 15 days of light treatment, the color index of each plant was measured from 10 to 11 a.m. For hormone quantification and transcriptomic and metabolomic analyses, the third through eighth true leaves (six leaves in total) were collected from five pepper seedlings of each treatment. Samples were then mixed, divided into four separate tubes, quickly frozen in liquid nitrogen, and stored at −80°C.

### Quantification of photosynthetic parameters and agronomic traits

The effects of light intensity on yield and other agronomic traits of yellowed mutant pepper were measured in unfiltered light and 50% shading light treatment in the field. Each treatment cultivated 50 plants and each treatment was repeated three times. Photosynthetic parameters were measured using an LI-6400XT portable photosynthesis meter (LI-COR, USA). From the first harvest, the fruit sets of plants were counted on days 7, 14, 19, 24, 29, and 34, respectively. The fruit of capsicum that grew to the mature green stage was picked to measure single fruit weight and calculate the yield. The total yield was calculated according to the average planting of 2000/667m^2^ plants in Hunan province.

### Quantification of plant color

Leaf color index was determined using a Ts7600 spectrophotometer (Shenzhen ThreeNH Technology). Chlorophyll and xanthophyll concentrations were quantified using HPLC in the absence of light and at low temperatures. Samples were ground in liquid nitrogen, and 1 g of crushed sample was added to a 5-ml brown volumetric flask. A 1-ml volume of 0.1% butylated hydroxytoluene (BHT)–ethanol solution was added to the crushed sample, and an additional 1 ml of 0.1% BHT–ethanol solution was used to rinse the sample. The final volume in the flask was adjusted to 5 ml with BHT–ethanol, and the entire flask was placed in a constant temperature oscillator at room temperature for 4 hours. After extraction, the flask was set at room temperature for 5 minutes. The 1-ml extract was filtered through a 0.22-μm filter and transferred to a 2-ml brown injection bottle. HPLC analysis was performed using a VWD/DAD detector, a C18 (250 × 4.6 mm; 5 μl) chromatographic column, an injection volume of 10 μl, 445 nm, and a flow rate of 1.0 ml/min at 25°C. The mobile phase used was 1 (methanol:water 88:12) and 0.1% methyl *tert*-butyl ether (80:20, v/v).

### Extraction, identification, and quantification of carotenoids

Leaf samples were freeze-dried and ground to powder with an MM 400 mixing grinder (Retsch) and zirconia beads (30 Hz, 1 min). Fifty milligrams of dried powder was extracted with a 1:2:1 (v/v/v) solution of *n*-hexane, acetone, and ethanol. The extraction solutions were swirled at room temperature for 20 minutes and centrifuged at 12 000  rpm for 6 minutes before the supernatant was collected. The extraction steps above were repeated once more, and the supernatants were combined. The extract was concentrated and redissolved in a 3:1 (v/v) mixture of methanol and methyl *tert*-butyl ether. Finally, the supernatant was collected for adsorption filtration (pore size, 0.22 μm) and stored in a brown sample vial for LC–MS/MS analysis.

Standard solutions of plant carotenoids were prepared at a range of concentrations, the peak intensities of their corresponding quantitative signals were obtained, and standard curves were constructed for each carotenoid. The absorbance peak areas for all samples were calculated using a linear equation created from the standard curves, and the absolute carotenoid content in each sample was calculated as $\left(C\times V\right)/\left(1000\times M\right)$, where *C* is the concentration obtained by comparing the area of the integrated value of the absorbance peak to the standard curve (μg/ml), *V* is the sample volume (μl), and *M* is the sample mass (g). The metabolomic analysis was performed by Metware Biotechnology (Wuhan, China).

### Hormone quantification

Plant hormones were detected and quantified by LC–MS/MS (Metware). Frozen samples were ground to powder with an MM400 ball mill (Retsch) operating at 30 Hz for 1 minute. Fifty milligrams of powder was transferred to a 2-ml tube, frozen in liquid N_2_, and dissolved in 1 ml of a 15:4:1 (v/v/v) solution of methanol, water, and formic acid. Ten microliters of a 100 ng/ml internal standard was added to the extract; the mixture was vortexed for 10 minutes, then centrifuged at 12 000 rpm and 4°C for 5 minutes. The supernatant was transferred to a clean tube and evaporated to dryness. The remaining solid was dissolved in 100 μl of 80% methanol and filtered through a 0.22-μm membrane filter for LC–MS/MS analysis.

### RNA extraction, library preparation, sequencing, and RNA-seq analysis

A complete description of the methods used for RNA extraction, library preparation, sequencing, and analysis can be found in our previous publication [49]. In brief, total RNA was extracted from pepper leaves, and at least three biological replicates were collected and mixed. The integrity, purity, and concentration of the purified RNA were evaluated using agarose gel electrophoresis and a spectrophotometer. Sequencing and assembly were carried out by Biomarker Technology (Beijing, China). The cDNA library was constructed and sequenced on the Illumina NovaSeq 6000 system, and clean reads were mapped to the reference genome of *C. annuum* (L_Zunla-1) (https://solgenomics.net/ftp/genomes/Capsicum_annuum/C.annuum_zunla/). The number of mapped reads and transcript length were standardized, and the transcript abundance was quantified as FPKM. The edgeR package was used to identify DEGs using a false discovery rate-adjusted *P*-value of <.01 and a fold change of ≥2. GO and KEGG (Kyoto Encyclopedia of Genes and Genomes) enrichment was performed as described previously [50, 51]. Heat maps and Venn diagrams were created using TBtools software [52].

### Subcellular localization of *bHLH71-like*

The *bHLH71-like* coding sequence (CDS) without a stop codon was ligated into *pCAMBIA1300* to obtain *pCAMBIA1300-bHLH71-like-GFP*. The recombinant plasmid was transformed into *Agrobacterium* strain GV3101 by the heat shock method. *pCAMBIA1300-bHLH71-like-GFP* and the *HY5-mCherry* nuclear marker were then co-transformed into leaves of *N. benthamiana*. After 2 days, fluorescence was observed and imaged using an LSM 800 confocal laser scanning microscope (Zeiss, Germany).

### Yeast one-hybrid assays

The Matchmaker Yeast One-Hybrid Library Screening System (Clontech, USA) was used for yeast one-hybrid assays. The *bHLH71-like* CDS was amplified and ligated into *pGADT7* to obtain *pGADT7-bHLH71-like*, and the *CaVDE* promoter fragment was amplified and ligated into *pHIS2.1* to obtain *pHIS2.1-pCaVDE*. 3-Amino-1,2,4-triazole (3-AT) was added to the selection medium to reduce yeast growth owing to leaky expression from *pHIS2.1*. Strain Y187 yeast harboring *pHIS2.1-pCaVDE* and pGADT7 or *pGADT7-bHLH71-like* were spotted onto synthetic dropout (SD)/−Trp/−Leu medium and SD/−Trp/−Leu/−His medium. Strains harboring *pHIS2-P53* and *AD-Rec2-P53* served as a positive control.

### Dual-luciferase reporter assays

The CDS of *bHLH71-like* was amplified and ligated into *pGreenII-62-SK*, and the *bHLH71-like* promoter was amplified and ligated into *pGreenII-0800-LUC* by using the ClonExpress II One Step Cloning Kit (Vazyme, USA). The constructs were transferred into *Agrobacterium* strain GV3101 (pSoup-p19) to generate *62SK-bHLH71-like* and *pCaVDE-LUC* recombinant strains. *Agrobacterium* strains were co-infiltrated into *N. benthamiana* leaves in a 9:1 ratio of strains harboring a transcription factor to strains harboring a promoter driving luciferase expression. Plants were placed in a growth chamber for 2 days to allow transient gene expression to occur. After 2 days, the tobacco leaves were harvested for fluorescence signal observation, and luminescence from firefly and *Renilla* luciferases was detected with the Dual-LUC Reporter Assay System (Promega). A Tanon 4600SF imaging system was used to collect fluorescence signal images. Two leaf disks (2 cm diameter) were ground in 500 ml of Passive Lysis Buffer, and 8 μl of crude extract was added to 40 μl of Luciferase Assay Buffer. Promoter activity was measured by calculating the LUC/REN ratio on a Promega GloMax 20/20 luminometer. Specific primers are provided in Supplementary Data Table S4. Three independent biological experiments were performed.

### Virus-induced gene silencing

Full-length CDSs of *CaVDE* and *bHLH71-like* were obtained from the NCBI database (https://www.ncbi.nlm.nih.gov). Specific target sites for silencing were selected using the VIGS tool through the Sol Genomics Network (https://vigs.solgenomics.net/), and specific primers for amplifying the target regions in *CaVDE* and *bHLH71-like* were designed using Geneious Prime software (Supplementary Data Table S4). The *pTRV2* vector was digested with EcoRI and BamHI and recovered, the purified product of the target fragment was recombined with the *TRV2* vector, and the recombined product was transformed into *Escherichia coli* DH5α. The recombined bacterial solution was spread on a kanamycin-resistant plate to select a single clone. We Performed PCR identification and *HR-TRV2* primer sequencing verification; extracted and identified the correct virus recombinant plasmid DNA to transform the *Agrobacterium* strain GV3101; obtained the *Agrobacterium* containing the recombinant vector; carried out PCR identification on the *Agrobacterium* single clone with the specific primers of the target gene; and prepared the infection solution (10 mM MgCl_2_, 10 mM MES, 200 uMAS). Plants were infected by the leaf injection infection method, and empty vector and reporter gene (phytoenedesaturase, *PDS*) were used as controls. Pepper plants were placed in the dark for 24 hours after inoculation, and then the silenced plants were transferred to a 16-hour light (200umol/m2/s, 20 ± 2°C)/8-hour dark (18 ± 2°C) photoperiod with 70% relative humidity in an artificial climate incubator. Three weeks after infiltration, some plants of the *TRV-PDS* positive control began to grow white leaves, indicating that VIGS had effectively inoculated the pepper plants. Specific primers *TRV1* and *HR-TRV2* were used to detect the new leaves of the plants inoculated with TRV:*CaVDE* and TRV:*bHLH71-like* (Supplementary Data Table S4). Finally, the identified positive plants of the target gene and the control were treated with high light (500 μmol/m^2^/s).

### Quantitative real-time PCR

qRT–PCR was performed as described in Taylor *et al*. [53] using the Vazyme fluorescent quantitative kit (ChamQ Universal SYBR qPCR Master Mix, Jiangsu, China) with cDNA as a template. The real-time fluorescence quantitative reaction volume was 20 μl, and the method of adding samples was as described in the the kit instructions. Gene-specific primers for qPCR were designed based on selected sequences from RNA-seq (Supplementary Data Table S1). Relative gene expression was calculated using the 2^−ΔΔCt^ method [54].

### Statistical analysis

Unless otherwise noted, results are expressed as mean ± standard error and were analyzed in Excel 2010 and SPSS 23.0. Duncan’s test at a significance level of *P* < .05 was used to determine whether means were significantly different.

## Acknowledgements

This research was funded by the Special Project of Biological Seed Industry and Fine and Deep Processing of Agricultural Products (grant 202202AE090031), the Project of Education Department of Hunan Province (grant 22B0229), and the Key Research and Development Program of Hainan Province (grant ZD2020060).

## Author contributions

Conceptualization, S.Y. and L.O.; methodology, Z.L., B.Y. and L.M.; formal analysis, Z.L. and L.M.; investigation, Y.D., X.L., and Y.C.; writing the original draft, Z.L. and L.M.; review and editing of the draft, X.D. and S.Y.; visualization, Z.L. and Q.C.; funding acquisition, X.Z. and L.O.

## Data availability

RNA-seq data generated in this study are available at the NCBI Sequence Read Archive (http://www.ncbi.nlm.nih.gov/sra) under accession number PRJNA771934.

## Conflict of interest

None declared.

## Supplementary data

Supplementary data are available at *Horticulture Research* online.

## Supplementary Material

Web_Material_uhad098
