# Supplementary figures and images for: A multi-omics approach identifies bHLH71-like as a positive regulator of yellowing leaf pepper mutants exposed to high-intensity light
Source: Hortic Res. 2023 May 12;10(7):uhad098. doi: 10.1093/hr/uhad098 (PMC10323627; doi:10.1093/hr/uhad098)

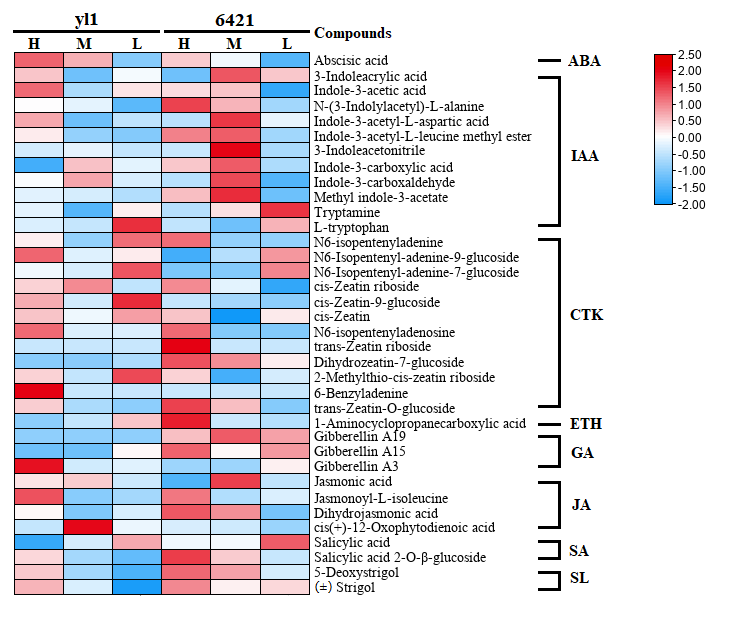

Supplement: Web_Material_uhad098 [file web_material_uhad098.zip › Fig.S1.png]
